# Translation initiation factor eIF3 promotes programmed stop codon readthrough

**DOI:** 10.1093/nar/gkv421

**Published:** 2015-04-29

**Authors:** Petra Beznosková, Susan Wagner, Myrte Esmeralda Jansen, Tobias von der Haar, Leoš Shivaya Valášek

**Affiliations:** 1 Laboratory of Regulation of Gene Expression, Institute of Microbiology ASCR, Videnska 1083, Prague 142 20, the Czech Republic; 2 Faculty of Science, Charles University, Vinicna 5, Prague 128 44, the Czech Republic; 3 School of Biosciences, University of Kent, Kent CT2 7NJ, UK

## Abstract

Programmed stop codon readthrough is a post-transcription regulatory mechanism specifically increasing proteome diversity by creating a pool of C-terminally extended proteins. During this process, the stop codon is decoded as a sense codon by a near-cognate tRNA, which programs the ribosome to continue elongation. The efficiency of competition for the stop codon between release factors (eRFs) and near-cognate tRNAs is largely dependent on its nucleotide context; however, the molecular mechanism underlying this process is unknown. Here, we show that it is the translation initiation (not termination) factor, namely eIF3, which critically promotes programmed readthrough on all three stop codons. In order to do so, eIF3 must associate with pre-termination complexes where it interferes with the eRF1 decoding of the third/wobble position of the stop codon set in the unfavorable termination context, thus allowing incorporation of near-cognate tRNAs with a mismatch at the same position. We clearly demonstrate that efficient readthrough is enabled by near-cognate tRNAs with a mismatch only at the third/wobble position. Importantly, the eIF3 role in programmed readthrough is conserved between yeast and humans.

## INTRODUCTION

Terminating protein synthesis at the appropriate stop codon is as important as initiating at the proper start codon. During canonical termination the stop codon is decoded by the release factor 1 (eRF1) that enters the A-site of the 80S ribosome in complex with the GTP-binding protein eRF3 (reviewed in (1)). In eukaryotes, eRF1 recognizes all three stop codons (UAA, UAG and UGA) with high precision. However, in some specific cases, not all stop codons signal the proper end of translation, which can thus continue beyond to the next stop codon. Generally speaking, translation termination can be viewed as a competition between stop codon recognition by release factors and stop codon decoding by near-cognate tRNAs. This competition differs genome-wide in its efficiency. The efficiency can be influenced by the identity of the stop codon (2–4), the nucleotide context of the stop codon (5–7), the identity of the last two amino acids incorporated into the polypeptide chain (8), the identity of the P-site tRNA (9) and the presence of stimulatory elements downstream from the stop codon (10–12). All these features increase the odds of the stop codon being decoded by a near-cognate, natural suppressor tRNA rather than by eRF1, resulting in the process termed stop codon readthrough. This allows production of C-terminally extended polypeptides with new or at least modified biological roles compared to their shorter, original versions. The mechanism whereby near-cognate tRNAs outcompete conventional stop codon recognition by eRF1 is not yet known, nor is which protein factors, if any, might be functionally important for stop codon readthrough.

In recent years several groups have proposed that the stop codon readthrough mechanism is specifically regulated by cis-acting RNA elements downstream of the first stop codon that may exist to generate proteome diversity in response to changing environmental conditions. The rapidly growing list of cellular genes under the control of this ‘programmed stop codon readthrough’ mechanism, the typical, long standing example of which is from the tobacco mosaic virus (TMV) genome (11), strongly suggests that programmed stop codon readthrough is an important contributor to general translational control in all kingdoms of life (for review; see (13–16)). A recent ribosome profiling study detected many readthrough events occurring at biologically relevant levels in budding yeast, fruit fly and human data sets, suggesting that this mechanism is highly conserved (17).

We recently reported that, besides the well-known eukaryotic release factors eRF1 and eRF3, translation termination is also regulated by the initiation factor eIF3 and the eIF3-associated protein HCR1, at least in yeast (18). eIF3 is a large multi-subunit protein complex that orchestrates numerous initiation steps in close cooperation with other eIFs (for review see (19,20)) and has been shown to interact with the solvent-exposed side of the 40S ribosomal subunit covering both mRNA entry and exit channels (21–27). We have demonstrated that besides the small ribosomal subunit, eIF3 also associates with the 80S termination complex in an eRF1-dependent manner and, by a yet to be elucidated mechanism, controls stop codon readthrough as several eIF3 mutations decrease its relative efficiency *in vivo*, showing antagonistic effects with mutations in both eRFs (18).

In this study we present data strongly suggesting that eIF3 is a crucial factor promoting programmed stop codon readthrough and describe the molecular mechanism of its action. eIF3, upon binding to programmed pre-termination complexes; i.e. pre-termination complexes on stop codons in unfavorable termination contexts, interferes with decoding of the third stop codon position and thus shifts the equilibrium toward stop codon readthrough by near-cognate tRNAs. As a result, it allows near-cognate tRNAs with a mismatch at the third position to decode the stop codon as a sense codon and continue with polypeptide synthesis. We show that eIF3 acts specifically on genes containing the aforementioned stimulatory elements downstream from the stop codon that are known to promote programmed stop codon readthrough and that this eIF3 function is evolutionary conserved.

## MATERIALS AND METHODS

### Yeast strains and plasmids

The lists and descriptions of plasmids and yeast strains used throughout this study (summarized in Supplementary Tables S1–S3) can be found in the Supplementary Data.

### Stop codon readthrough assays

**Figure 1.**
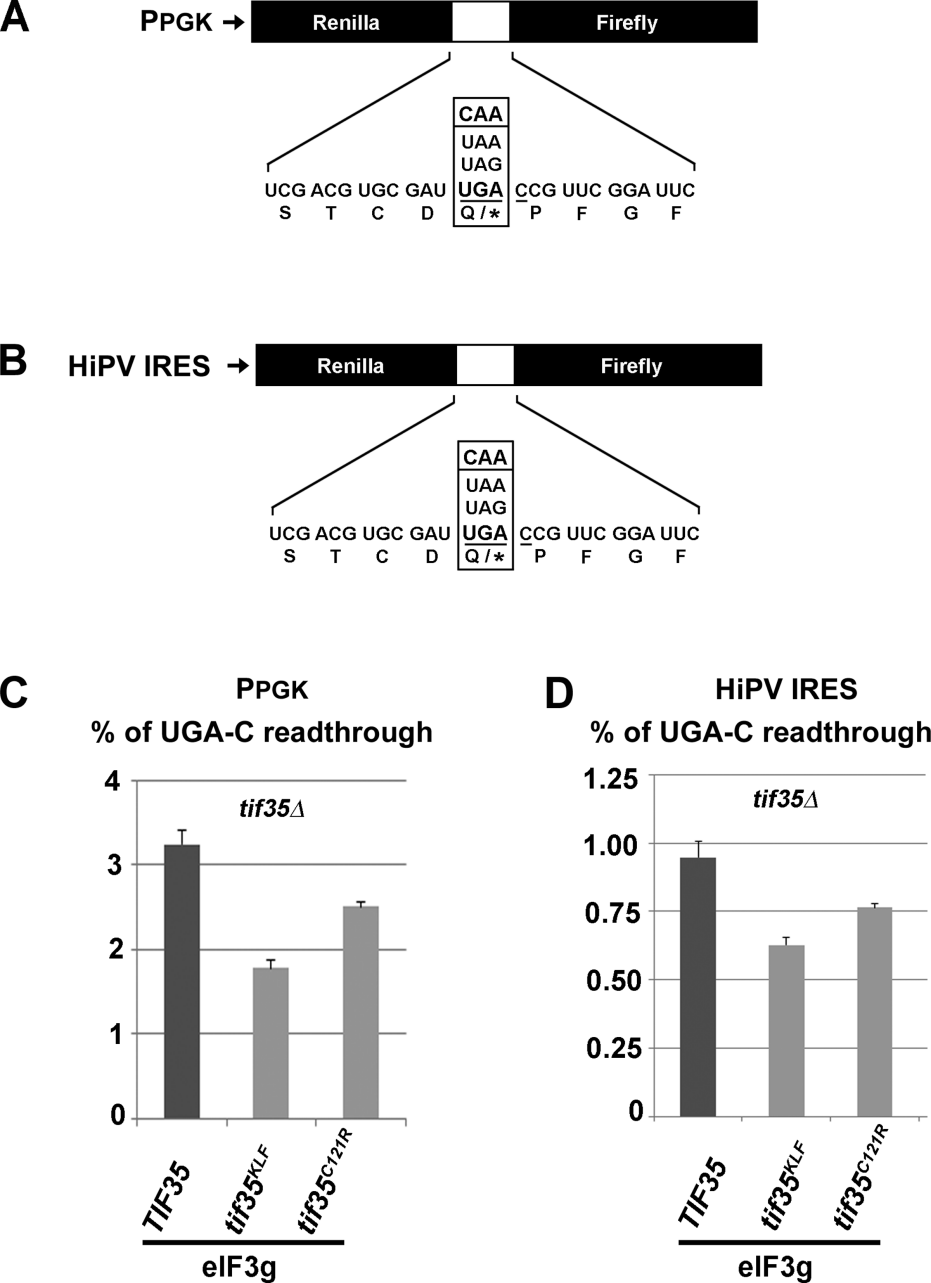
Readthrough measurements using the standard dual luciferase assay are not sensitive to defects in translation initiation. (**A**) Schematics of the standard dual luciferase readthrough reporter constructs with variable stop codons (or a CAA coding codon) under the P_PGK_ promoter adapted from (29). (**B**) Schematics of the modified dual luciferase readthrough reporter constructs containing the HiPV IRES in their 5′ UTRs. (**C**) eIF3 mutations affect stop codon readthrough independently of their initiation phenotypes. The *TIF35* wt, *tif35-KLF* and *tif35-C121R* mutant alleles were introduced into the H464 strain by plasmid shuffling. The resulting transformants were grown in SD and processed for stop codon readthrough measurements using standard dual luciferase readthrough reporter constructs schematically illustrated in panel A as described in Materials and Methods. (**D**) Same as in panel C except that modified dual luciferase reporter constructs YEp-HiPV-UGAC-L and YEp-HiPV-CAAC-L, schematically illustrated in panel B, were employed.

The majority of stop codon readthrough assays in this study were performed using a standard bicistronic reporter construct (Figure 1A) bearing a *Renilla* luciferase gene followed by an in-frame firefly luciferase gene. Separating the two genes is either a tetranucleotide termination signal (UGA-C) or, for control purposes, the CAA sense codon followed by cytosine. In indicated cases the termination signal and/or the following nucleotide context was modified. It is noteworthy that this system avoids possible artifacts associated with changes in the efficiency of translation initiation associated with the the nonsense mediated decay (NMD) pathway (28), because both *Renilla* and firefly enzymes initiate translation from the same AUG codon. For further details please see (29). The experiments and data analysis were carried out according to the microtiter plate-based dual luciferase protocol developed by (30) and commercially distributed by Promega. The samples were processed in quintuplicates and each experiment was repeated at least three times.

Stop codon readthrough measurements with newly developed dual luciferase constructs driven by the HiPV IRES (Figure 1B) were performed essential the same as described above except that the transformed cells were not directly grown in microtiter plates but in Erlenmeyer flasks in 25 ml of the SD media to OD–0.6. The whole cell extracts (WCEs) were prepared in 1× phosphate buffered saline (PBS) supplemented with 1% Triton X-100 using FastPrep Instrument (MP Biomedicals) at the intensity level of 5 in one 40 seconds long cycle. The 10-fold diluted WCEs were then subjected to the aforementioned microtiter plate-based protocol according to the vendor's instruction.

Stop codon readthrough assays in HeLa cells were performed as follows. Cells were grown in 24-well plates in Dulbecco's modified eagle's medium (DMEM) (Sigma; catalog no. D6429) supplemented with 10% fetal bovine serum (FBS) (Sigma; catalog no. F7524). Twenty-four hours after seeding, the cells were transfected with ON-TARGETplus small interfering RNA (siRNA) cocktail systems from Dharmacon/Thermo Scientific (human eIF3a, catalog no. L-019534-00; human eIF3g, catalog no. L-019533-00; non-targeting siRNA, catalog no. D-001810-10) at concentration of 5nM. INTERFERin (Polyplus; catalog no. 409) was used as a transfection reagent and transfections were carried out according to the vendor's instructions (3 μl/well). After 24 h the media was changed. Transfection of the readthrough reporter plasmids was carried out 48 h after the siRNA transfection using TurboFect (Thermo Scientific; catalog no. R0531), according to the vendor's instructions (500 ng of plasmid DNA and 1 μl of Turbofect per each well) in triplicates. One day after this second transfection, the cells were washed in 1× PBS and lysed directly on plate with 1xGlo lysis buffer (Promega; catalog no. E266A). Equal amounts of each lysate were then transferred into two white flat-bottom 96-well plates. One plate was treated with the Bright-Glo Luciferase Assay System (Promega; catalog no. E2620) to measure the firefly luciferase signal according to the vendor's instructions. The other plate was treated with the Renilla-Glo Assay System (Promega; catalog no. E2720/50). Subsequently, the ratio of the firefly to renilla luciferase signal was calculated for each reporter (bearing either UAG or CAG). The actual readthrough value (in%) was obtained as the ratio of the normalized UAG value to the normalized CAG value. This experiment was repeated three times.

### Polysomal gradient analysis

The 0.5% formaldehyde (HCHO) cross-linking followed by WCE preparation and fractionation of WCEs for analysis of translational complexes were carried out as described previously (18,31) with the following exceptions. Cycloheximide was added at a concentration of 0.05 mg/ml 5 min before the HCHO treatment, after which the cells were instantly broken by the FastPrep Instrument (MP Biomedicals) at the intensity level of 5 in two consecutive 20 s cycles. The resulting WCEs were separated on 5–45% sucrose gradients.

## RESULTS

### Readthrough measurements using the standard dual luciferase assay are not sensitive to defects in translation initiation

A standard way to measure the efficiency of stop codon readthrough is by dual-luciferase reporter assays specifically designed to be independent of mRNA level changes (29). In this reporter system, two genes encoding luciferases are separated by an in frame stop codon or by the CAA coding triplet as a reference (Figure 1A). To increase the sensitivity of this assay, the UGA stop codon with the C at the fourth position (UGA-C), which is known to allow relatively high levels of readthrough, is typically used (5). Using this approach, we previously demonstrated that eIF3 controls translation termination and stop codon readthrough; mutations in the core subunits of eIF3 decrease the relative efficiency of readthrough with this pre-sensitized construct (18). However, this reporter system is, by definition, dependent on the canonical initiation event on the first luciferase gene. In order to investigate whether the involvement of eIF3 in termination is fully independent of its role in initiation, we replaced the 5′ UTR of the first luciferase gene in our reporters with the cricket paralysis virus IRES-like HiPV IRES (Figure 1B). This IRES allows initiation of translation in the absence of any initiation factors (32–34) and, importantly, was shown to initiate translation also in the yeast *Saccharomyces cerevisiae*. We then employed these first-canonical-initiation-event-independent constructs to re-examine the readthrough efficiency of our eIF3 mutants and observed similar effects when compared to the standard reporter; stop codon readthrough is sensitive to eIF3 mutants in both cases (Figure 1C and D; *P* < 0.05 for both measurements of both mutants). These results not only completely rule out any impact of the initiation phenotypes of our eIF3 mutants on their termination phenotypes, but also fully justify the use of these standard readthrough reporters for subsequent analysis of the role of eIF3 in termination and stop codon readthrough.

### eIF3 promotes stop codon readthrough but only on stop codons in an unfavorable termination context

**Figure 2.**
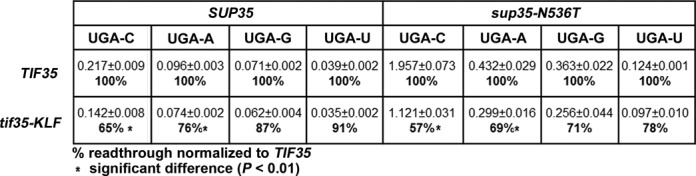
Wild-type eIF3 promotes stop codon readthrough but only on stop codons in the unfavorable termination context. The *TIF35* wt and *tif35-KLF* mutant alleles were introduced into the PBH140 (*tif35Δ SUP35*) and PBH134 (*tif35Δ sup35-N536T*) strains by plasmid shuffling. The resulting transformants were grown in SD and processed for stop codon readthrough measurements as described in Materials and Methods. Percentages of readthrough of *tif35-KLF* given in the table were calculated from measured values (mean ± SE; *n* = 6) normalized to *TIF35* wt. Changes in readthrough levels were analyzed by the student's *t*-test and shown to be statistically significant only for those values marked with the asterisk (*P* < 0.01).

Previous studies of readthrough implicated the nucleotide context surrounding the stop codon in modulating readthrough rates in various systems (3,5–7). Since eIF3 controls stop codon readthrough, we asked whether or not its action is also dependent on the stop codon nucleotide context. Because termination efficiency is particularly sensitive to the nucleotide immediately following the stop codon (5,6), we employed bicistronic reporters (kindly provided by D. Bedwell) bearing the in-frame UGA stop codon between both luciferases containing all possible combinations of the ‘stop’ tetranucleotide; i.e. UGA-C with the most efficient readthrough (5), UGA-A, -G or –U (Figure 2). Firstly, we observed that the impact of the fourth nucleotide on the relative termination efficiency of the UGA-N tetranucleotide was gradual in the following sequence: C > A > G > U (from the least to the most efficient termination) (Figure 2). Secondly, the effect of *tif35-KLF* on readthrough also turned out to be gradual and, in addition, in perfect accord with the C > A > G > U sequence of impact. The greatest difference in termination efficiency between wild-type (wt) and mutant *TIF35* was seen with UGA-C (∼1.5-fold in *SUP35* genetic background), followed by the UGA-A tetranucleotide etc. (Figure 2; *SUP35*). The differences seen in UGA-G and -U were not statistically significant; see also below. These findings thus indicate that wt eIF3 increases readthrough but preferentially on stop codons in unfavorable termination contexts—readthrough values change from the lowest in UGA-U *tif35-KLF* (0.035) to the highest in UGA-C *TIF35* (0.217; i.e. 6.2-fold), with UGA-C *tif35-KLF* reaching only 0.142 (4.1-fold). In other words, readthrough sensitive eIF3 mutations prevent full propagation of stop codon readthrough that the wt eIF3 complex is normally capable of—in the context-dependent manner—by a molecular mechanism elucidated below.

To corroborate these results in a pre-sensitized genetic background with higher basal levels of readthrough, we performed the same type of experiment in the *sup35-N536T* mutant, which increases readthrough by up to ∼9-fold, and observed essentially the same effects (Figure 2; *sup35-N536T*). The differences between *TIF35* wt and mutant cells detected in UGA-G and –U cases remained statistically insignificant, even though the individual readthrough values measured with these constructs are much higher than those in *SUP35* wt cells, practically as high as those seen with UGA-C and UGA-A in *SUP35* wt cells (Figure 2; compare *SUP35* and *sup35-N536T*). This suggests that the observed effects of wt eIF3 are dependent on the identity of the fourth nucleotide (C or A) and not simply on the absolute level of readthrough.

### eIF3 stimulation of stop codon readthrough is dependent on its association with pre-termination 80S complexes *in vivo*

We showed previously that eIF3 core subunits associate with 80S couples isolated from heavy polysomes and that this association is dependent on the presence of termination factors eRF1 and eRF3, implying that these eIF3-bound 80S species most probably represent the pre-termination 80S complexes (pre-TCs) (18). To investigate how eIF3 mutations prevent its participation in stop codon readthrough, we first asked whether or not eIF3 mutations interfere with its ability to associate with the pre-TCs.

**Figure 3.**
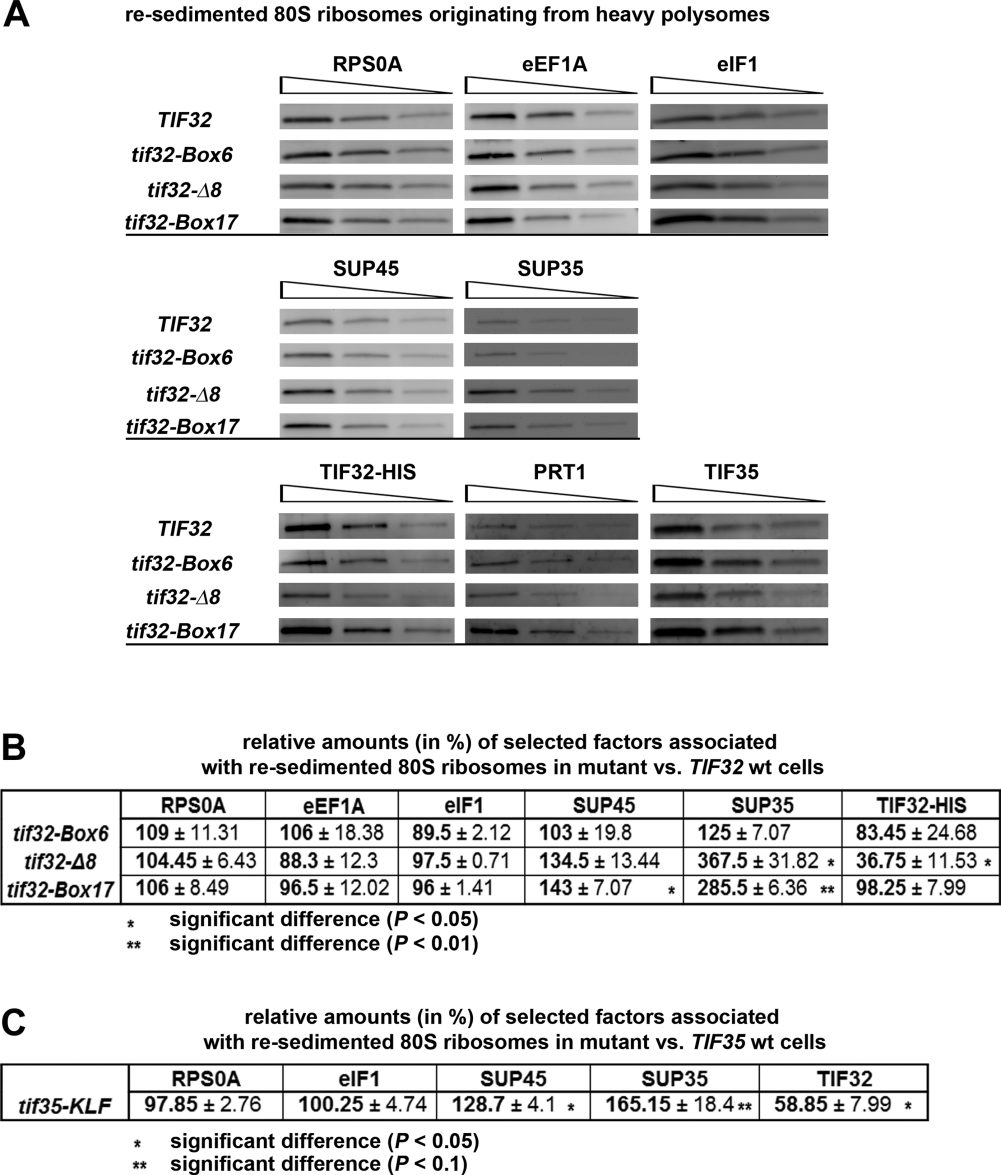
eIF3 stimulation of stop codon readthrough is dependent on its association with pre-termination 80S complexes *in vivo*. (**A**) Changes in amounts of translation factors associated with the pre-TCs in cells bearing *TIF32* mutations *in vivo*. Plasmid-born mutant alleles of *TIF32* (*tif32-Box6, tif32-Δ8* and *tif32-Box17*) and its corresponding wild*-*type (*TIF32*) were introduced into YBS52 strain by plasmid shuffling. The resulting transformants were grown in SD medium at 30°C to an OD_600_ of ∼1 and cross-linked with 0.5% HCHO prior to harvesting. WCEs were prepared, separated on a 5–45% sucrose gradient by centrifugation at 39 000 rpm for 2.5 h and heavier polysome fractions were collected and treated with RNase I to separate the pre-initiation complexes (PICs) from 80S couples on actively translated mRNAs. Thus treated samples were subjected to the second sucrose gradient centrifugation according to the resedimentation protocol described before and 80S couples—predominantly containing termination complexes (18)—were collected, loaded in six two-fold dilutions onto the SDS-PAGE gel and processed for western blot analysis with antibodies raised against factors shown above the strips. Three representative dilutions for each factor are shown. (**B**) Quantification of selected factors from at least three independent experiments shown in Figure 3A with corresponding *P*-values, where applicable. Western blot signals from each of the six two-fold dilutions obtained with individual anti-bodies were quantified by NIH ImageJ and plotted against their corresponding loadings. Individual slopes (representing relative amount of each factor in mutant cells) calculated from the linear regression of resulting plots were normalized to the slope obtained with the *TIF32* wt strain, which was set to 100%. (**C**) Changes in amounts of translation factors associated with the pre-TCs in cells bearing the *tif35-KLF* mutation *in vivo*. Quantification of selected factors from at least three independent experiments carried out as described in panel A with the exception that *tif35-KLF* and its corresponding *TIF35* wt were introduced into the H464 strain by plasmid shuffling.

To do that, we selected three mutants of the a/TIF32 subunit of eIF3 and analyzed the amounts of factors bound to the 80S couples isolated from heavy polysomes using the resedimentation protocol described previously (18,31). Briefly, WCEs derived from formaldehyde cross-linked cells were resolved on sucrose gradients after high velocity centrifugation. The higher polysomal fractions were collected, treated with RNase I and the resulting 43S–48S pre-initiation complexes and 80S species were separated in a second round of centrifugation (resedimentation). Isolated 80S couples were loaded in six serial two-fold dilutions onto sodium dodecyl sulphate-polyacrylamide gel electrophoresis and the amounts of the small ribosomal protein uS2/RPS0A (as a loading control) and 80S-associated factors were analyzed by quantitative western blotting. As can be seen in Figure 3A and B, mutations *tif32-Δ8* and *tif32-Box17*, two mutant eIF3 complexes that fail to fully promote readthrough (18), displayed statistically significant enrichment of the eRF1 (*SUP45*) and eRF3 (*SUP35*) signal in heavy polysomes compared to the wt control. In addition to that, in the case of the stronger *tif32-Δ8* mutant, there is a visible reduction (by ∼60%; Figure 3B) in the amounts of selected eIF3 subunits bound to pre-TCs in comparison to wt. Similar results were also obtained for another strong terminating mutant (*tif35-KLF*) in the g/TIF35 subunit of eIF3 (Figure 3C). Together these results suggest that heavy polysomes in these three specific mutants contain a significantly higher proportion of terminating ribosomes lacking eIF3. Importantly, initiation and elongation factors eIF1 and eEF1A, respectively, neither of which imparts any termination phenotype when down-regulated (18), showed no significant alterations (Figure 3A and B; residual amounts of eIF1 occurring in the 80S samples most probably reflect the existence of an eIF1-containing ‘eIF3-translasome’ complex proposed to associate with elongating 80S ribosomes (35)—we used it as a specificity control). These observations further underscore our conclusion that the observed termination efficiency changes specifically arise from eIF3 association with the pre-TCs and not with initiating or elongating ribosomes. Furthermore, our observation that the amount of ribosome-associated eEF1A remains unchanged also indicates that eIF3 mutants do not impact the elongation phase. Taken together with the fact that the *tif32-Box6* mutant—which does not affect translation termination (18)—did not produce any significant difference in factor occupancy in the pre-TCs (Figure 3A and B), these results suggest that the presence of eIF3 within the pre-TCs is an important requirement for its stimulatory role in stop codon readthrough.

### eIF3 is one of the major players in programmed stop codon readthrough

As aforementioned, UGA-C belongs to a group of tetranucleotides that are under-represented in genomes and that allow relatively inefficient termination (5) (Figure 2). Another sequence-specific feature implicated in permitting even less efficient termination than UGA-C; i.e. promoting much higher readthrough, is the hexanucleotide CAR-NBA consensus sequence immediately following the stop codon (10). This ‘readthrough element’ is exploited by several viral and cellular genes to extend their C-termini in a specific regulatory mechanism referred to as ‘programmed stop codon readthrough’ (13–15).

**Figure 4.**
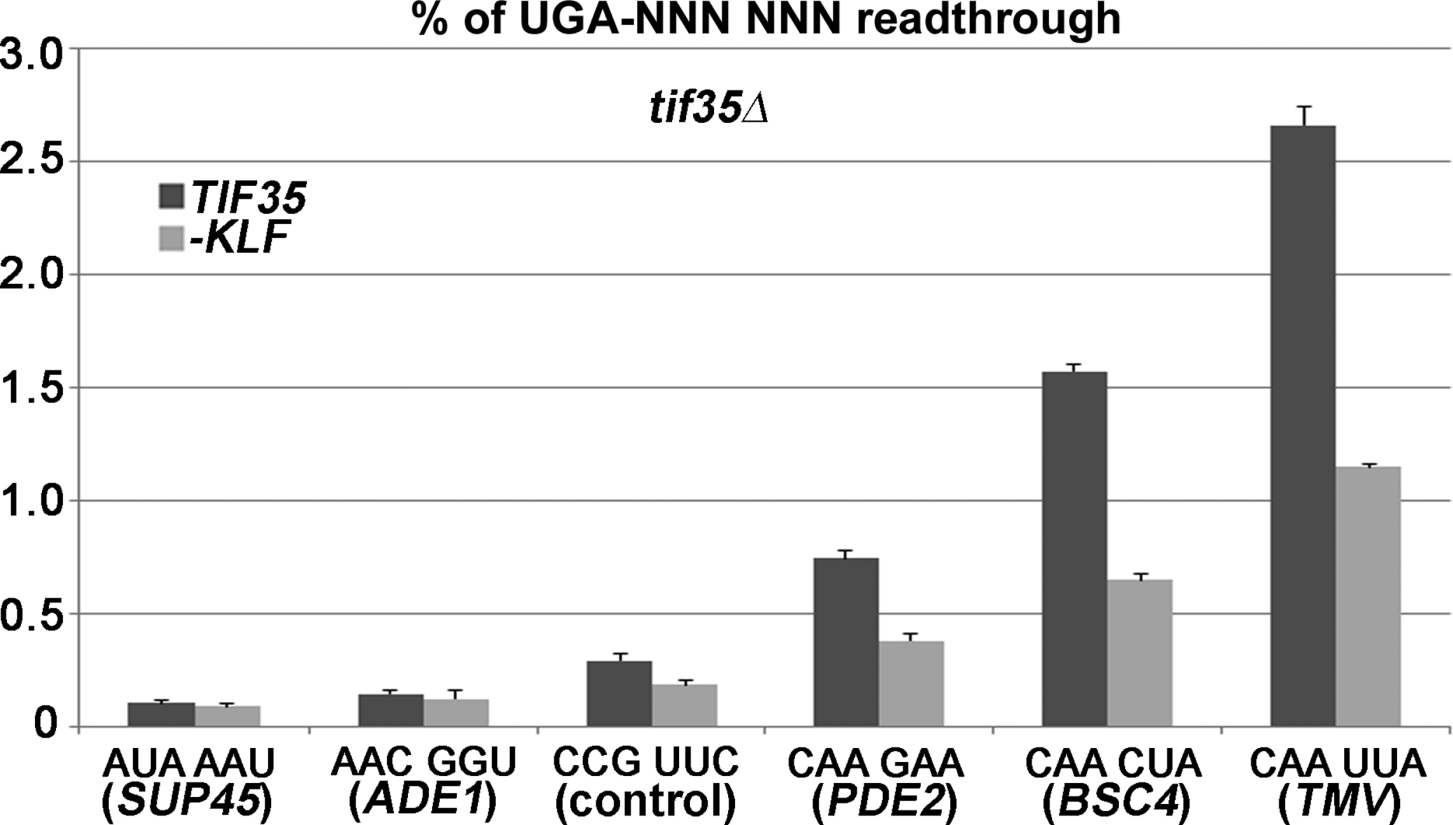
eIF3 is one of the major players in programmed stop codon readthrough. The PBH140 derivatives bearing *TIF35* wt and *tif35-KLF* mutant alleles (generated as described in Figure 2) were grown and processed for stop codon readthrough measurements as described in Materials and Methods, except that besides our standard (‘control’) pTH477 readthrough construct (shown in Figure 1A), five derivatives with variable hexanucleotide sequence immediately following the stop codon were also employed: PBB85 (for *SUP45*), PBB83 (for *ADE1*), PBB84 (for *PDE2*), PBB80 (for *BSC4*) and PBB82 (for *TMV*).

To explore whether the ability of eIF3 to promote readthrough specifically on poor-context stop codons is important also for this specific ‘programmed’ regulatory mechanism, we replaced the hexanucleotide sequence immediately following the stop codon of our UGA-C standard reporter with several pre-selected hexanucleotide sequences. In particular, UGA-CCG-UUC from our elevated-readthrough-promoting reporter (Figure 1A), which we took as a positive control, was exchanged for UGA-AUA-AAU or UGA-AAC-GGU, representing the hexanucleotides taken from the *SUP45* and *ADE1* termination sequences, respectively. These were deliberately chosen as a negative control because of their highly efficient termination. In the next three replacements we used termination hexanucleotides from genes known to undergo programmed stop codon readthrough, the strongest of which is the CAA-UUA hexanucleotide of the TMV (11). Among cellular genes from the *S. cerevisiae* genome, we picked *BSC4* (CAA-CUA) and *PDE2* (CAA-GAA). The hexanucleotide of *BSC4* is very similar in sequence to the TMV hexanucleotide and, consistent with this, it was classified as one of the genes with the highest level of stop codon bypass efficiency (36). In the case of *PDE2*, its hexanucleotide was directly implicated in affecting its biological role via increased readthrough (37). As can be seen in Figure 4, all three modified reporters containing the CAR-NBA or CAR-NNA consensus sequences produced robust readthrough, the efficiency of which is strictly dependent on intact eIF3 bound to pre-TCs (compare *TIF35* wt versus mutant datasets). In contrast, the inefficient readthrough observed for both negative controls is practically wt eIF3-independent. These results suggest that eIF3 is one of the major players in programmed stop codon readthrough.

### Wild-type eIF3 promotes incorporation of aminoacyl-tRNAs with a mismatch at the third position of the stop codon

**Figure 5.**
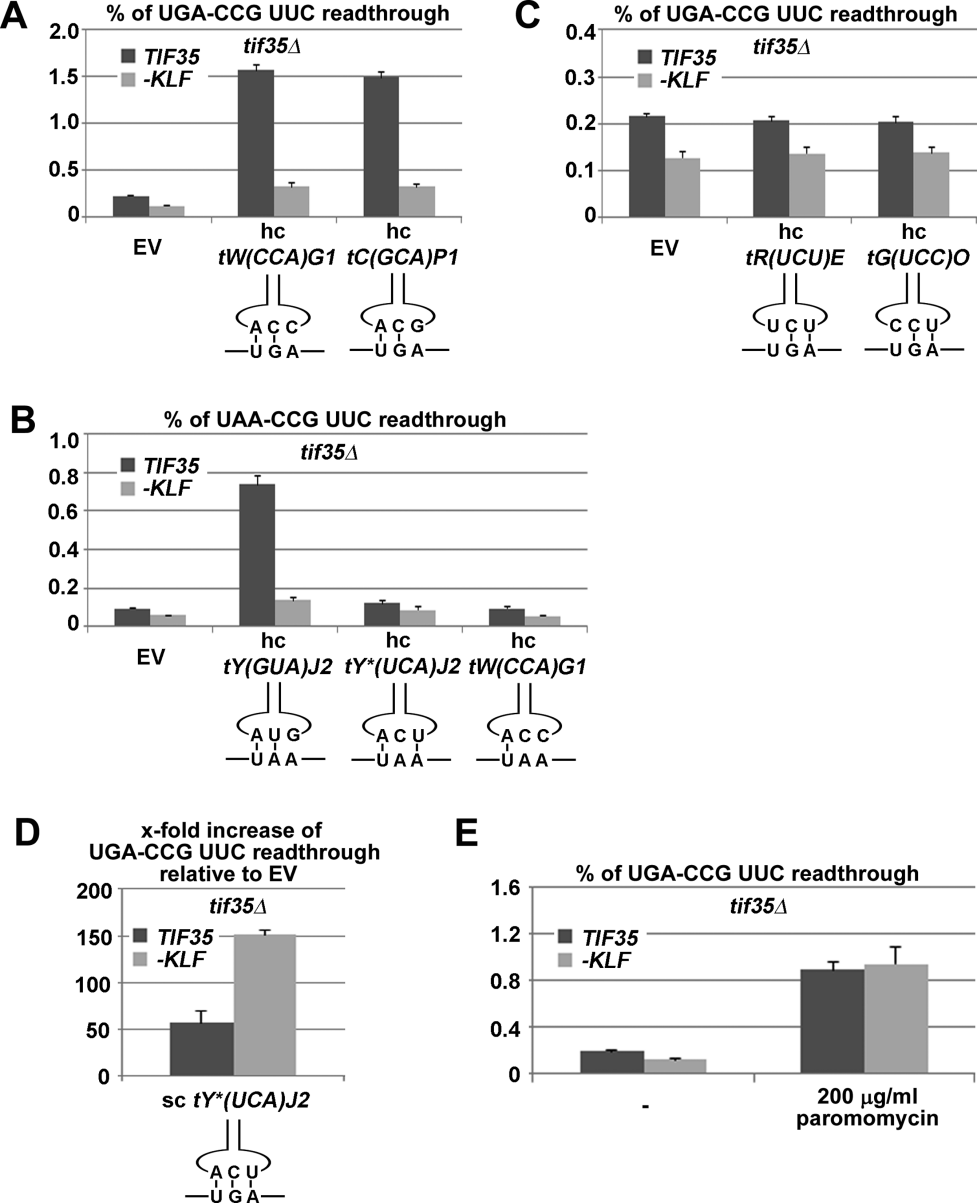
eIF3 interferes with decoding of the third position of the stop codon allowing incorporation of near-cognate tRNAs with the mismatch at the same position. (**A**) eIF3 promotes incorporation of both existing near-cognate tRNAs with a mismatch at the third position of the UGA stop codon. The PBH140 derivatives bearing *TIF35* wt and *tif35-KLF* mutant alleles (generated as described in Figure 2) were transformed with either empty vector (EV), high copy (hc) tW(CCA)G1 or hc tC(GCA)P1 and the resulting transformants were grown and processed for stop codon readthrough measurements as described in Materials and Methods. (**B**) eIF3 enhances incorporation of the near-cognate tRNA also at the UAA stop codon with a mismatch at the third position [tY(GUA)J2—encoding tyrosine] but not with a mismatch at the second position [tY*(UCA)J2] or non-cognate [tW(CCA)G1—encoding tryptophan]. The PBH140 derivatives bearing *TIF35* wt and *tif35-KLF* mutant alleles (generated as described in Figure 2) were transformed with empty vector (EV), hc tY(GUA)J2, hc tY*(UCA)J2 or hc tW(CCA)G1 and subsequently also with the readthrough construct YEp-R/T-UAAC-L and the resulting transformants were grown and processed for stop codon readthrough measurements as described in Materials and Methods. (**C**) eIF3 does not affect incorporation of both existing near-cognate tRNAs with a mismatch at the first position of the UGA stop codon. The PBH140 derivatives bearing *TIF35* wt and *tif35-KLF* mutant alleles (generated as described in Figure 2) were transformed with either empty vector (EV), hc tR(UCU)E or hc tG(UCC)O and the resulting transformants were grown and processed for stop codon readthrough measurements as described in Experimental Procedures. (**D**) eIF3 impedes stop codon decoding by fully cognate tRNA. The PBH140 derivatives bearing *TIF35* wt and *tif35-KLF* mutant alleles (generated as described in Figure 2) were transformed with either empty vector (EV) or a single copy (sc) plasmid carrying tY*(UCA)J2 and the resulting transformants were grown and processed for stop codon readthrough measurements as described in Materials and Methods. (**E**) Paromomycin nullifies the effect of wt eIF3 on programmed stop codon readthrough. The PBH140 derivatives bearing *TIF35* wt and *tif35-KLF* mutant alleles (generated as described in Figure 2) were grown in SD without or with 200μg/ml paromomycin for 6 h and processed for stop codon readthrough measurements as described in Materials and Methods.

Next we wished to uncover the molecular mechanism by which eIF3 promotes programmed stop codon readthrough. Considering that readthrough occurs when the stop codon is decoded by near-cognate tRNAs (i.e. tRNAs that can base-pair with two of the three stop codon bases) (16), we first explored which aminoacyl-tRNAs get incorporated during programmed stop codon readthrough. We did so by measuring readthrough using programmed reporters in cells individually overexpressing selected near-cognate tRNAs versus control empty vectors. We expected that overexpression of a near-cognate tRNA that successfully decodes the corresponding programmed stop codon will increase readthrough levels significantly more in *TIF35* wt cells than in *tif35-KLF* mutant cells, which are seriously compromised in promoting programmed stop codon readthrough (Figure 4). In particular, we tested (i) all known near-cognate tRNAs that form Watson–Crick base pairs with the first 2 nt of the UGA stop codon [tW(CCA) and tC(GCA)] as well as of both UAA and UAG stop codons [tY(GUA)]; (ii) both existing near-cognate tRNAs that form Watson–Crick base pairs with the second and third positions of the UGA stop codon [tR(UCU) and tG(UCC)]; (iii) a mutated tyrosine tRNA [tY*(UCA)] unable to base pair with the second position of the UAA stop codon only; and (iv) several non-cognate tRNAs that form Watson–Crick base pairs only with the first position of either UAA [tW(CCA)] or UAG [tY*(UCA) and tW(CCA)] stop codons chosen as negative controls. As can be seen in Figure 5, wt eIF3 promotes readthrough of the UGA stop codon by incorporation of tryptophan [tW(CCA)] or cysteine [tC(GCA)] tRNAs (Figure 5A) with a mismatch at the third position, and also of the UAA and UAG stop codons by incorporation of the tyrosine [tY(GUA)] tRNA with the same mismatch (Figure 5B and Supplementary Figure S1). In contrast, overexpression of near-cognate or non-cognate tRNAs with a single mismatch either at the first or second positions, respectively, did not significantly increase readthrough in eIF3 wt cells (Figure 5B and C), similarly to overexpression of our control, non-cognate tRNAs base-paring only with the first stop codon nucleotide (Figure 5B and Supplementary Figure S1). These results suggest that wt eIF3 specifically promotes incorporation of near-cognate tRNAs with a mismatch at the third (wobble) position of a given programmed stop codon. In addition, these results also imply that the effect of eIF3 on programmed stop codon readthrough is not limited to the UGA stop codon alone, as it promotes readthrough on all three stop codons and to the same degree, provided that they are set in a readthrough-permissive context.

### Wild-type eIF3 interferes with decoding of the third (wobble) position of programmed stop codons

Taking into account that wt eIF3 promotes read-though by only those near-cognate tRNAs with a mismatch at the third (wobble) position of the programmed stop codon, we wondered whether it may play a more direct role in decoding of the third position *per se*. We overexpressed tY*(UCA), a tyrosine-coding tRNA in which the anti-codon has been mutated to the cognate of UGA, assuming that if eIF3 specifically causes mis-decoding at the wobble position (which would be beneficial for incorporation of the above defined near-cognate tRNAs), the UGA stop codon suppressing impact of this artificial cognate tRNA would be more pronounced in *TIF35* mutant versus wt cells. Indeed, overexpressing tY*(UCA) results in a ∼150-fold increase in UGA readthrough in *tif35-KLF* mutant cells compared to a ∼50-fold increase in eIF3 wt cells (Figure 5D). Together these results imply that wt eIF3 does generally impair decoding of the third (wobble) position of a stop codon. Thus it is highly likely that it directly interferes with the stop codon recognition process by the eRF1.eRF3.GTP complex, as we recently proposed (18). This particular effect of eIF3 would play only a negligible role during the canonical termination event (Figure 4); however, it would become critical in programmed stop codon readthrough as a necessary prerequisite for the subsequent incorporation of near-cognate tRNAs with the mismatch at the wobble position (see our model below).

### The miscoding agent paromomycin, disabling ribosomal discrimination against near-cognate tRNAs, nullifies the effect of wt eIF3 on programmed stop codon readthrough

The aminoglycoside antibiotic paromomycin is a widely used drug for termination studies as it has been shown that its presence within the pre-TCs causes displacement of the A1493 phosphate group relaxing the A-site codon decoding pocket. As a result, paromomycin changes the deformation of the near-cognate codon-anticodon helix, after which the ribosome does not actively sense the correct Watson–Crick base-pairing geometry and thus does not discriminate against near-cognate tRNAs (38). Because wt eIF3 was proposed to act similarly on poor-context stop codons, we re-examined the efficiency of UGA-C readthrough in *TIF35* wt versus mutant cells in the presence of 200 μg/ml paromomycin. Consistent with previous results, we observed increased readthrough efficiency in the presence of paromomycin (Figure 5E). In addition, no difference in the efficiency of readthrough promoted by either wt or mutant g/TIF35 was observed in the presence of paromomycin, perhaps because the magnitude of the paromomycin effect masks that of eIF3 or because eIF3 no longer promotes readthrough if the decoding center is perturbed.

### The role of eIF3 in stop codon readthrough is conserved between yeast and humans

**Figure 6.**
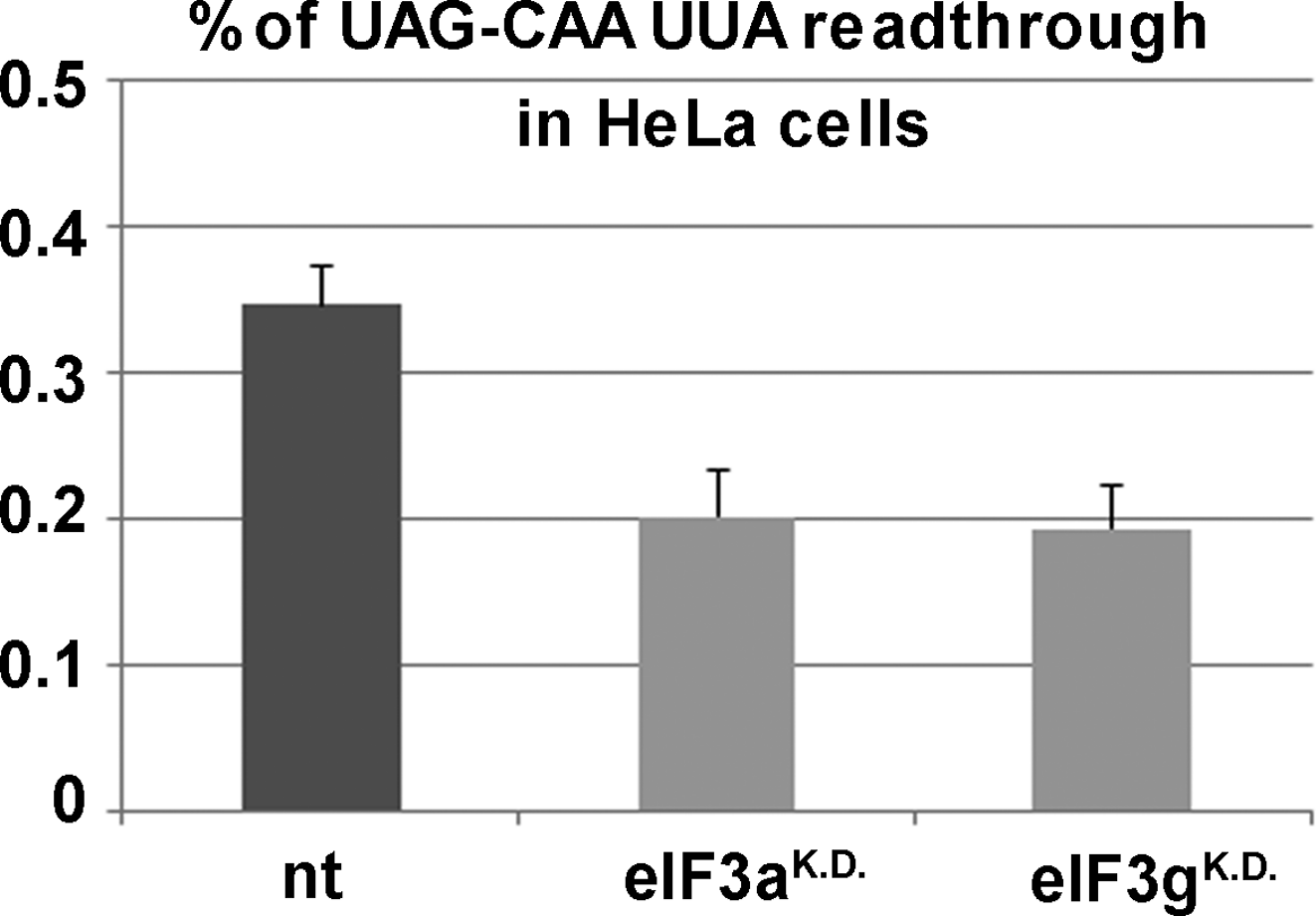
Human eIF3 promotes stop codon readthrough. HeLa cells were transfected with siRNA against the eIF3a and eIF3g subunits (eIF3a^K.D.^ and eIF3g^K.D.^) or non-targeting siRNA (nt) as control. Two days later, the siRNA-treated cells were further transfected with the bicistronic firefly/*renilla* luciferase readthrough reporters (40). Both luciferases are separated by a stop (UAG) or sense codon (CAG) followed—in both cases—by a highly readthrough permissive sequence (CAAUUA) from TMV (41). The UAG-C readthrough is displayed as % of the signal obtained with the sense codon reporter; standard deviations are given.

Because programmed stop codon readthrough has been shown to play a critical role in regulating gene expression in several organisms such as plant viruses, yeast, fruit flies, and mammals, and because the number of genes apparently under the control of this regulatory mechanism continues to increase (see for example the most recent genome-wide high-throughput screen (17)), we wished to investigate whether the role of eIF3 in programmed stop codon readthrough is evolutionarily conserved. To do that, we individually knocked down the a (eIF3a^K.D.^—homolog of yeast *TIF32*) and g (eIF3g^K.D.^—homolog of yeast *TIF35*) subunits of human eIF3 in HeLa cells using the ON-TARGETplus SMART pool siRNA system (Dharmacon/Thermo Scientific) following the previously established protocol (39). On the second day after the transfection of these specific siRNAs and a control non-targeting siRNA (nt), the cells were further transfected with human dual-luciferase reporter plasmids containing the highly permissive TMV CAA-UAA hexanucleotide sequence immediately following the stop codon to boost the sensitivity of this assay (40,41). As can be seen in Figure 6, downregulation of both human eIF3 subunits resulted in a significant decrease of the efficiency of readthrough, to a similar degree as that previously observed with mutations in orthologous eIF3 subunits in yeast (18) (Figure 1C). This finding suggests that the role of eIF3 in termination, and in particular in stop codon readthrough, has been conserved throughout evolution.

## DISCUSSION

The proteomic diversity achieved in all kingdoms of life, by various regulatory mechanisms, is extraordinary. Growing evidence suggests that stop codon readthrough is one mechanism whereby cells expand the functional repertoire of proteins beyond an apparently limited genetic script (17,37,42–44). For example, a C-terminal isoform of the endothelial growth factor A (VEGFA) generated by programmed readthrough has been shown to exhibit anti-angiogenic activity in contrast to the pro-angiogenic activity of the original protein (42); efficient programmed readthrough occuring at the UGA-CU termination hexanucleotide of two normally cytosolic enzymes was shown to target these new protein C-terminal isoforms into peroxisomes in both fungi and animals (44). Despite the growing importance of this paradigm, our mechanistic knowledge of how pre-termination ribosomes recognize a programmed stop codon for readthrough and how the readthrough event occurs on the molecular level is still rather poor.

Here, we demonstrate that the *bona fide* translation initiation factor eIF3, recently implicated in controlling translation termination besides its critical initiation roles (18), is one of the major trans-acting factors in programmed stop codon readthrough in yeast and perhaps also in mammals (Figure 6). In particular, we show that the presence of eIF3 in pre-TC complexes increases readthrough on stop codons in unfavorable termination contexts in a manner that is independent of its role during initiation (Figures 1–3). More importantly, eIF3 is critically required for efficient readthrough on three mRNAs previously demonstrated to be under the control of the programmed stop codon readthrough pathway (Figure 4). More detailed analysis of the codon specificity of eIF3-mediated stop codon readthrough suggests that eIF3 promotes the incorporation of near-cognate tRNAs with mismatches at the third (wobble) position, perhaps via an interaction with the decoding center of the ribosome.

Our previous experiments strongly suggested that the association of eIF3 with pre-TCs is strictly dependent on eRF1 (18). Thus it seems likely that eIF3 is generally present at any termination event regardless of its canonical or programmed readthrough predisposition, although we cannot rule out that it may preferentially associate with the readthrough programmed pre-TC by recognizing their unique structural features.

**Figure 7.**
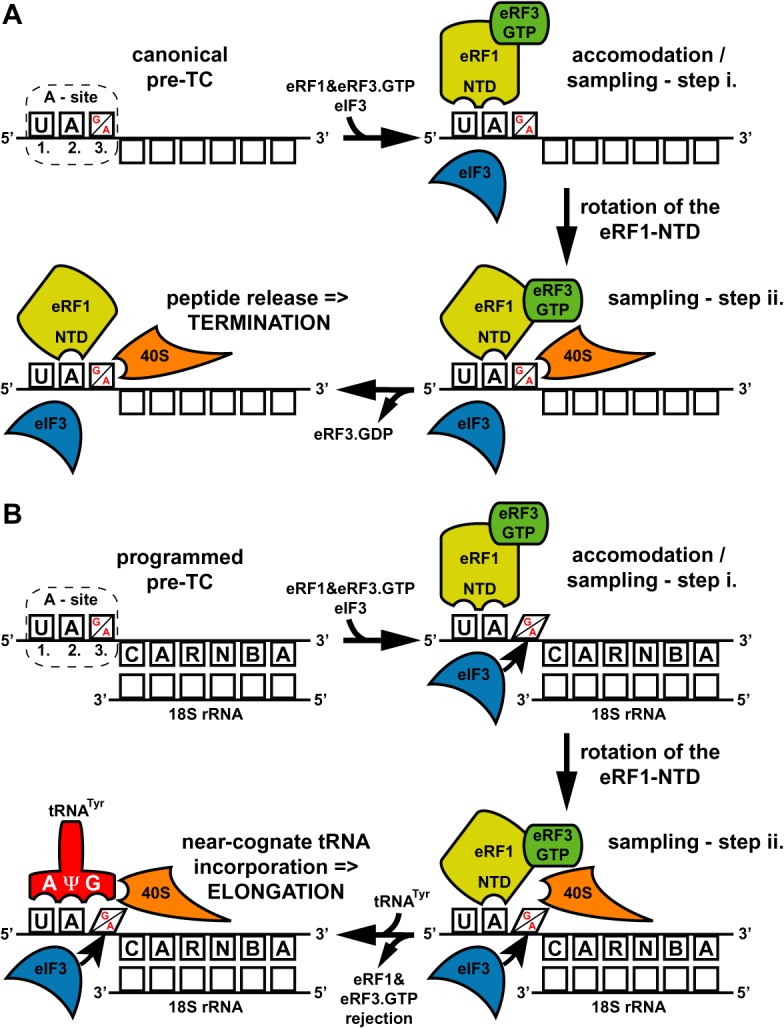
Model: translation initiation factor eIF3 promotes programmed stop codon readthrough. (**A**) Canonical termination; stop codon in the termination favorable context appears in the A-site (only UAG and UAA stop codons are indicated for illustration purposes; UGA works by the same mechanism), eRF1 in complex with eRF3.GTP binds to it and samples the codon in a two-step process including conformational re-arrangements of the eRF1-NTD. During the second step the ribosome by itself co-participates in this accommodation phase that ultimately leads to GTP hydrolysis on eRF3, polypeptide release and ribosomal recycling (see text for further details). (**B**) Programmed stop codon readthrough; stop codon occurs in the unfavorable termination context bearing specific consensus sequences like CAR-NBA in its 3′ UTR—in this particular case proposed to base-pair with 18S rRNA. The eIF3 presence in the pre-TC—perhaps in co-operation with these sequences—alters the decoding property of the nucleotide at the third stop codon position. This prevents its proper decoding during the second sampling step and subsequently, after the eRF1-eRF3.GTP complex rejection, allows incorporation of near-cognate tRNAs with the mismatch at the third position to read through the stop codon and continue with elongation.

During canonical termination (Figure 7A), when the stop codon in the termination favorable context appears in the ribosomal A-site, eRF1, in complex with eRF3.GTP, enters the A-site triggering major conformational re-arrangements of the pre-TC, particularly at and around the mRNA entry channel (45–47). During these ribosome-specific re-arrangements, the N-terminal domain (NTD) of eRF1 itself undergoes an important conformational change, which plays a critical role in the two-step model of stop codon selection (sampling), as recently proposed by (48). In the first step, the first and second nucleotides of the stop codon are recognized by specific residues of the eRF1-NTD (Figure 7A, ‘sampling—step i.’), followed by its re-arrangement during the second step, which permits decoding of the third nucleotide. It has been proposed that—as a result of this re-arrangement—the second stop codon nucleotide is contacted by different amino acid residues of the eRF1-NTD than during the first step; the third nucleotide is most probably recognized directly by the ribosome, highly likely in close cooperation with eRF1 (Figure 7A, ‘sampling—step ii.’). (An alternative option is that the ribosome recognizes the second nucleotide and the third is read by eRF1 – for simplicity, our model in Figure 7 depicts only the former option.) As a result, eRF1 stably accommodates in the A-site triggering GTP hydrolysis on eRF3, followed by downstream termination/recycling events. The presumed presence of eIF3 in the pre-TCs with stop codons in a favorable termination context would have only a negligible, if any, impact on termination efficiency (Figure 4).

During programmed readthrough (Figure 7B), the stop codon occurs in an unfavorable termination context that promotes stop codon readthrough. One example of this is the CAR-NBA hexanucleotide immediately following the stop codon. The effect of this and other, functionally similar sequences varies. They can for example base-pair with 18S rRNA to stabilize and/or specifically rearrange the pre-TC, such as in the CAR-NBA case (10). Alternatively, they can recruit trans-acting factors proposed to interact with translating ribosomes in a way that prevents eRF1 binding to the stop codon as in the very recently reported case of vascular endothelial growth factor A (VEGFA) mRNA (42). Because a part of the eIF3 body projects into the vicinity of the mRNA entry channel and several of its subunits interact with RNA (reviewed in (19), see also (24,27,49–50)), it is conceivable that eIF3 could directly interact with these sequences and perhaps even mediate their effects in promoting programmed readthrough; however, there is no experimental evidence for that yet. In light of our results, we propose that during or after the first step of the two-step recognition mechanism, which would happen uninfluenced by eIF3 (single mismatches at the first or second stop codon positions showed no genetic interaction with eIF3 whatsoever—Figure 5B and C), eIF3 alters the decoding property of the third stop codon nucleotide. It could do this either directly, as it was proposed to reach the A-site via its a/TIF32 and c/NIP1 subunits (50,51) and interact with the N-M domain of eRF1 via its g/TIF35 subunit (18) or allosterically by binding to constituents of the decoding pocket such as the small ribosomal protein uS5/RPS2. Indeed, uS5/RPS2 was shown to interact with the C-terminus of a/TIF32 (25) and, remarkably, act as an omnipotent suppressor of all three stop codons while bearing the *SUP44* mutation (52). Either way, as a result, proper decoding of the third stop codon position by the ribosome and/or eRF1 would be impaired, as indicated in Figure 5D, resulting in premature rejection of the eRF1.eRF3.GTP complex from pre-TCs. Subsequently, the eIF3-mediated altered property of the third position would invite near-cognate tRNAs with a mismatch at this very position (tyrosine tRNA for UAA or UAG and tryptophan and cysteine tRNAs for UGA) to incorporate into the A-site and allow elongation to continue to the next stop codon downstream (Figure 5A and B). This is consistent with the fact that paromomycin, which makes the pre-TCs incapable of discriminating against near-cognate tRNAs and thus dramatically increases readthrough, prevented eIF3 from further increasing readthrough over its mutant showing genetic epistasis (Figure 5E). Taken together, we propose that eIF3 disables proper decoding of the third stop codon position by the ribosome co-operating with the ‘cognate’ eRF1 and at the same time promotes wobble miscoding by near-cognate tRNAs to occasionally win the battle over the stop codon predisposed for programmed readthrough.

Programmed stop codon readthrough has recently emerged as an important contributor to proteomic diversity and has also been implicated in the replication of several pathogenic RNA virus families and thus represents an appealing target for antiviral therapies (53–56). Programmed stop codon readthrough has also been proposed as a potential treatment for hereditary diseases caused by premature termination codons (PTCs), many of which occur in unfavorable termination contexts (for review see (57)). PTCs produce truncated proteins that are non-functional or even toxic and targeted, mRNA-specific increase in readthrough could restore the functionality of the affected genes and thus cure the disease, provided that the exact molecular mechanism is known and can be purposefully exploited. Here, we reveal that the translation initiation factor eIF3 plays a crucial role in programmed stop codon readthrough and suggest a detailed molecular model for its role. Our results thus open new avenues of inquiry for understanding this important biological event also with respect to human health.

## SUPPLEMENTARY DATA

Supplementary Data are available at NAR Online.

SUPPLEMENTARY DATA
