# Supplementary material for: Translation initiation factor eIF3 promotes programmed stop codon readthrough
Source: Nucleic Acids Res. 2015 Apr 29;43(10):5099–111. doi: 10.1093/nar/gkv421 (PMC4446449; doi:10.1093/nar/gkv421)
Supplement: SUPPLEMENTARY DATA [file supp_43_10_5099__index.html]

Translation initiation factor eIF3 promotes programmed stop codon readthrough — Translation initiation factor eIF3 promotes programmed stop codon readthrough — SUPPLEMENTARY DATA 

# Translation initiation factor eIF3 promotes programmed stop codon readthrough

## SUPPLEMENTARY DATA

**Files in this Data Supplement:**

- SUPPLEMENTARY DATA
