# Supplementary material for: Translation initiation factor eIF3 promotes programmed stop codon readthrough
Source: Nucleic Acids Res. 2015 Apr 29;43(10):5099–111. doi: 10.1093/nar/gkv421 (PMC4446449; doi:10.1093/nar/gkv421)
Supplement: SUPPLEMENTARY DATA [file supp_gkv421_nar-00814-r-2015-File009.pdf]

SUPPLEMENTARY DATA

MATERIALS AND METHODS

Construction of yeast strains and plasmids

List of all strains used throughout this study can be found in Table S1.

To create PBH140 and PBH134 strains; 74D-694 and L2334, respectively, were first transformed with YCp11-TIF35-MET to cover for the deletion of *TIF35* that was made in the next step by introducing the *Bam*HI-*Aat*I 5.2kb fragment carrying the *tif35Δ::hisG-URA3-hisG* integration cassette from pΔtif35#9. The Uracil auxotrophy was regained by growing the cells on SD plates containing 5-fluoro-orotic acid (5-FOA). The resulting strain was subsequently transformed with YEp-TIF35-U and the Leucine auxotrophy was regained by growing the cells in liquid media containing Leucine and selecting for those that lost the YCp11-TIF35-MET plasmid on SD +/- Leucine plates producing PBH140 and PBH134.

List of all plasmids and PCR primers used throughout this study can be found in Tables S2 and S3, respectively.

pTH779 was created as follows. Nucleotides 6210-6505 of the viral genome, comprising the translational start at nucleotide 6473, were amplified from a cDNA clone of the viral RNA (1) using primers HiPV_Bam_5 and HiPV_FLuc_3. Firefly luciferase DNA was amplified from plasmid pTH650 (2) using primers Ff_5 and Ff_Sal_3. These sequences were then recombined using overlap extension PCR, by using the two PCR products as templates in a second round PCR reaction with primers HiPV_Bam_5 and Ff_Sal_3. This generates an in-frame fusion of the first 11 codons of the HiPV capsid protein, an XbaI restriction site, and firefly luciferase from codon 2. The recombination product was cloned as a BamHI/SalI fragment into pTH645 (2) to give pTH779.

YEp-HiPV-UGAC-L and YEp-HiPV-CAAC-L were constructed by three consecutive cloning steps. First, the 1959-pb *Xma*I-*Pst*I fragment from pTH779 was inserted into *Xma*I-*Pst*I digested YEp181. Next, the *Sph*I-*Alw*NI digested PCR product obtained with primers PBRFtermF and NIP1-AlwNI using pTH477 as a template was inserted into *Sph*I-*Alw*NI digested vector from previous step. Last, the *Not*I-*Pst*I digested PCR products obtained by PBRFpstI and PBRFNotI primers using pTH477 or YEp-R/T-CAAC-L as templates were inserted into *Not*I-*Pst*I digested vector from previous step resulting in YEp-HiPV-UGAC-L and YEp-HiPV-CAAC-L, respectively.

YEp-R/T-UAAC-L and YEp-R/T-UAGC-L were constructed by inserting the 4567-bp *Alw*NI-*Nsi*I fragment from pTH461 and pTH469, respectively, into YEplac181 digested by *Alw*NI-*Nsi*I.

PBB75, PBB76 and PBB77 were constructed by inserting the 4567-bp *Alw*NI-*Nsi*I fragment from pDB712, pDB714 and pDB716, respectively, into YEplac181 digested by *Alw*NI-*Nsi*I.

PBB80 was created by inserting the *Sal*I-*Not*I digested PCR product obtained with primers BSC4 and PBRFNotI using pTH477 as template into *Sal*I-*Not*I digested pTH477.

PBB82 was created by inserting the *Sal*I-*Not*I digested PCR product obtained with primers TMV and PBRFNotI using pTH477 as template into *Sal*I-*Not*I digested pTH477.

PBB83 was created by inserting the *Sal*I-*Not*I digested PCR product obtained with primers ADE1 and PBRFNotI using pTH477 as template into *Sal*I-*Not*I digested pTH477.

PBB84 was created by inserting the *Sal*I-*Not*I digested PCR product obtained with primers PDE2 and PBRFNotI using pTH477 as template into *Sal*I-*Not*I digested pTH477.

PBB85 was created by inserting the *Sal*I-*Not*I digested PCR product obtained with primers SUP45 and PBRFNotI using pTH477 as template into *Sal*I-*Not*I digested pTH477.

pTH335 was created by inserting the *BamHI-XhoI* digested PCR product obtained with primers tW_f and tW_r using genomic DNA obtained from yeast strain BY4741as template into *BamHI-XhoI* digested pRS426

pTH638 and pTH640 were created by inserting the *BamHI-HindIII* digested PCR products obtained with primers SUP4_f and SUP4_r using genomic DNA obtained from yeast strain BY4741 (for pTH638) or suppressor strain MT422/1c (for pTH640) as template into *BamHI-HindIII* digested pRS316.

PBB90 and PBB91 were constructed by inserting the 235-bp *Bam*HI-*Kpn*I fragment from pTH638 and pTH640, respectively, into pTH335 digested by *Bam*HI-*Kpn*I.

PBB97 was created by inserting the *BamHI-XhoI* digested PCR product obtained with primers PB94 and PB95 using genomic DNA obtained from yeast strain H464 as template into *BamHI-XhoI* digested pTH335.

PBB99 was created by inserting the *BamHI-XhoI* digested PCR product obtained with primers PB98 and PB99 using genomic DNA obtained from yeast strain H464 as template into *BamHI-XhoI* digested pTH335.

PBB100 was created by inserting the *BamHI-XhoI* digested PCR product obtained with primers PB100 and PB101 using genomic DNA obtained from yeast strain H464 as template into *BamHI-XhoI* digested pTH335.

Table S1. Yeast strains used in this study.

**Strain**	**Genotype**	**Source or reference**
YBS52 [a]	*MATa leu2-3, -112 ura3-52 trp1Δ gcn2Δ tif32Δ URA3::GCN2 ura3* (Ycp-a/TIF32-His-U)	(3)
H464[a]	*MATa leu2-3,-112 ura3-52::GCN2 trp1Δ tif35Δ* (hc *TIF35 URA3*)	(4)
PBH140[b]	*MATa ade1-14 trp1-289 his3-Δ200 leu2-3,112 ura3-52 tif35Δ* (YCp22-g/TIF35-screen)	this study
PBH134[b]	*MATa ade1-14 trp1-289 his3-Δ200 leu2-3,112 ura3-52 tif35Δ sup35-N536T* (YCp22-g/TIF35-screen)	this study
74D-694[b]	*MATa ade1-14 trp1-289 his3-Δ200 leu2-3,112 ura3-52*	(5)
L2334[b]	*MATa ade1-14 trp1-289 his3-Δ200 leu2-3,112 ura3-52 sup35-N536T*	(6)
BY4741	*MATa his3Δ0 leu2Δ0 met15Δ0 ura3Δ0*	(7)
MT422/1c	*ade2-1 his5-2 can1-100 ura3-1 leu2-3,122 lys1-1 met8-1 SUP4-o*	(8)

Table S2. Plasmids used in this study.

Plasmid	Description	Source of reference
pTH779	low copy HiPV-Firefly in *URA3* plasmid from pRS316	this study
YEp-HiPV-UGAC-L	high copy HiPV-Renilla-Firefly R/T cassette (stop codon of Renilla is UGA-C; for read-through measurements) in *LEU2* plasmid from YEplac181	this study
YEp-HiPV-CAAC-L	high copy HiPV-Renilla-Firefly R/T cassette (stop codon of Renilla is replaced with CAA-C [coding triplet]; for control read-through measurements) in *LEU2* plasmid from YEplac181	this study
YCp-a/TIF32-His-L	single copy wt *TIF32-His* in *LEU2* plasmid from YCplac111	(3)
YCp-a/tif32-Box17-His	single copy *tif32-Box17-His* in *LEU2* plasmid from YCplac111	(3)
YCp-a/tif32-Box6-His	single copy *tif32-Box6-His* in *LEU2* plasmid from YCplac111	(3)
YCp-a/tif32-Δ8-His-L	single copy *tif32-Δ8-His* in *LEU2* plasmid from YCplac111	Beznoskova et al 2013
YCp22-g/TIF35-screen	single copy wt *TIF35-His* in *TRP1* plasmid from YCplac22	(4)
YCp22-g/TIF35-KLF	single copy *TIF35-KLF-His* in*TRP1* plasmid from YCplac22	(4)
YCp22-g/TIF35-C121R	single copy *TIF35-C121R-His* in *TRP1* plasmid from YCplac22	(9)
YEp-R/T-UGAC-L	high copy PGK-Renilla-Firefly R/T cassette (stop codon of Renilla is UGA-C; for read-through measurements) in *LEU2* plasmid from YEplac181	(9)
YEp-R/T-CAAC-L	high copy PGK-Renilla-Firefly R/T cassette (stop codon of Renilla is replaced with CAA-C [coding triplet]; for control read-through measurements) in *LEU2* plasmid from YEplac181	(9)
YEplac181	high copy cloning vector, *LEU2*	(10)
YEplac195	high copy cloning vector, *URA3*	(10)
pSP35-45	high copy wt *SUP45 SUP35* in *URA3*	(11)
pTH335	high copy *URA3* vector (pRS426) containing genomic DNA surrounding the tW(CCA)G1 gene	this study
pTH461	high copy PGK-Renilla-Firefly R/T cassette (stop codon of Renilla is UAA-C; for read-through	(12)

	measurements) in *URA3* plasmid from YEplac195	
pTH469	high copy PGK-Renilla-Firefly R/T cassette (stop codon of Renilla is UAG-C; for read-through measurements) in *URA3* plasmid from YEplac195	(12)
YEp-R/T-UAAC-L	high copy PGK-Renilla-Firefly R/T cassette (stop codon of Renilla is UAA-C; for read-through measurements) in *LEU2* plasmid from YEplac181	this study
YEp-R/T-UAGC-L	high copy PGK-Renilla-Firefly R/T cassette (stop codon of Renilla is UAG-C; for read-through measurements) in *LEU2* plasmid from YEplac181	this study
pTH638	centromeric *URA3* vector (pRS316) containing the tY(GUA)J2 wild-type gene (=*SUP4*)	this study
pTH640	centromeric *URA3* vector (pRS316) containing an opal suppressor mutant of tY(GUA)J2 gene	this study
PBB90	high copy *URA3* vector (pRS426) containg the tY(GUA)J2 wild-type gene (=*SUP4*)	this study
PBB91	high copy *URA3* vector (pRS426) containing an opal suppressor mutant of tY(GUA)J2 gene	this study
PBB97	high copy *tC(GCA)P1* in *URA2* plasmid from pRS426	this study
PBB99	high copy *tR(UCU)E* in *URA2* plasmid from pRS426	this study
PBB100	high copy *tG(UCC)O* in *URA2* plasmid from pRS426	this study
pDB712	high copy PGK-Renilla-Firefly R/T cassette (stop codon of Renilla is UGA-A; for read-through measurements) in *URA3* plasmid from YEplac195	(12)
pDB714	high copy PGK-Renilla-Firefly R/T cassette (stop codon of Renilla is UGA-G; for read-through measurements) in *URA3* plasmid from YEplac195	(12)
pDB716	high copy PGK-Renilla-Firefly R/T cassette (stop codon of Renilla is UGA-U; for read-through measurements) in *URA3* plasmid from YEplac195	(12)

PBB75	high copy PGK-Renilla-Firefly R/T cassette (stop codon of Renilla is UGA-A; for read-through measurements) in *LEU2* plasmid from YEplac181	this study
PBB76	high copy PGK-Renilla-Firefly R/T cassette (stop codon of Renilla is UGA-G; for read-through measurements) in *LEU2* plasmid from YEplac181	this study
PBB77	high copy PGK-Renilla-Firefly R/T cassette (stop codon of Renilla is UGA-U; for read-through measurements) in *LEU2* plasmid from YEplac181	this study
pTH477	high copy PGK-Renilla-Firefly R/T cassette (stop codon of Renilla is UGA-CCGUUC; for read-through measurements) in *URA3* plasmid from YEplac195	(12)
PBB80	high copy PGK-Renilla-Firefly R/T cassette (stop codon of Renilla is UGA-CAACUA; for read-through measurements) in *URA3* plasmid from YEplac195	this study
PBB82	high copy PGK-Renilla-Firefly R/T cassette (stop codon of Renilla is UGA-CAAUUA; for read-through measurements) in *URA3* plasmid from YEplac195	this study
PBB83	high copy PGK-Renilla-Firefly R/T cassette (stop codon of Renilla is UGA-AACGGU; for read-through measurements) in *URA3* plasmid from YEplac195	this study
PBB84	high copy PGK-Renilla-Firefly R/T cassette (stop codon of Renilla is UGA-CAAGAA; for read-through measurements) in *URA3* plasmid from YEplac195	this study
PBB85	high copy PGK-Renilla-Firefly R/T cassette (stop codon of Renilla is UGA-AUAAAU; for read-through measurements) in *URA3* plasmid from YEplac195	this study
YCp11-TIF35-MET	single copy *TIF35* under *MET3* promoter, *LEU2* plasmid from Ycplac11	(4)
pΔtif35#9	the *TIF35* deletion construct with the *hisG-URA3-hisG* cassette inserted in	(4)

	between the 5‘ and 3‘ UTRs of *TIF35*	
YEp-TIF35-U	high copy wt *TIF35* in *LEU2* plasmid from YEplac352	(13)

Table S3. Primers used in this study.

**Primer name**	**Primer sequence (5´to 3´)**
HiPV_Bam_5	GCGCGGGATCCAAACATTGTGCGAAGCTTCTTGGCTC
HiPV_FLuc_3	CCTTTCTTTATGTTTTTGGCGTCTTCCTGCAGATTTGTATTGTTATTATTATT
Ff_5	GAAGACGCCAAAAACATAAAGAAAGG
Ff_Sal_3	GGGGGGGTCGACTTACAATTTGGACTTTCCGCCCTTC
PBRFtermF	AATAAGCATGCGCGGCCGCAAGCTTTTCGTGGCCGAGG
NIP1-AlwNI	CTGTTACCAGTGGCTGCTGCC
PBRFpstI	AATAACTGCAGACTTCGAAAGTTTATGATCCA
PBRFNotI	CTCGAAGCGGCCGCTCTAGAATTACAC
BSC4	CAAATGTCGACGTGCGATTGACAACTAGGATCCTTCAACTTCCCTGAGCTCG
TIF32	CAAATGTCGACGTGCGATTGACAGACAGGATCCTTCAACTTCCCTGAGCTCG
TMV	CAAATGTCGACGTGCGATTGACAATTAGGATCCTTCAACTTCCCTGAGCTCG
ADE1	CAAATGTCGACGTGCGATTGAAACGGTGGATCCTTCAACTTCCCTGAGCTCG
PDE2	CAAATGTCGACGTGCGATTGACAAGAAGGATCCTTCAACTTCCCTGAGCTCG
SUP45	CAAATGTCGACGTGCGATTGAATAAATGGATCCTTCAACTTCCCTGAGCTCG
tW_f	GCGCGCCTCGAGATTTTTTACATTTGTTCTATCAGTTAGT
tW_r	CGCGCGGGATCCTATAAAAAGAACATATTCATACGGGC
SUP4_f	CCCCCGGATCCTTCAATTGTATATGTGTTATGTAGTATAC
SUP4_r	CCCCCAAGCTTTTTCAACTTGCAAGTCTGGGAAGTG
PB94	AATAACTCGAGTTGCGTGGATAAGTGTTATTATTCTATTGCC
PB95	AATAAGGATCCAAAGCCGTACAGGCGAACGTATATAATTAAAATTC
PB98	AATAACTCGAGTAACACGTTAATATGGTGGAGTCAGCTGAG
PB99	AATAAGGATCCTAATCTGCCGTATGTTCTGGTATTTACTGGTTAGG
PB100	AATAACTCGAGTAATGTTATAGTGATTGAGATCAGTTTCACC
PB101	AATAAGGATCCGGTTATTTCACGTCCCGGAGGAAAAAAGTTTG

REFERENCES

1. Nakashima, N., Sasaki, J. and Toriyama, S. (1999) Determining the nucleotide sequence and capsid-coding region of himetobi P virus: a member of a novel group of RNA viruses that infect insects. *Archives of virology*, **144**, 2051-2058.
2. Chu, D., Barnes, D.J. and von der Haar, T. (2011) The role of tRNA and ribosome competition in coupling the expression of different mRNAs in Saccharomyces cerevisiae. *Nucleic Acids Res*, **39**, 6705-6714.
3. Munzarová, V., Pánek, J., Gunišová, S., Dányi, I., Szamecz, B. and Valášek, L.S. (2011) Translation Reinitiation Relies on the Interaction between eIF3a/TIF32 and Progressively Folded cis-Acting mRNA Elements Preceding Short uORFs. *PLoS Genet*, **7**, e1002137.
4. Cuchalová, L., Kouba, T., Herrmannová, A., Danyi, I., Chiu, W.-l. and Valášek, L. (2010) The RNA Recognition Motif of Eukaryotic Translation Initiation Factor 3g (eIF3g) Is Required for Resumption of Scanning of Posttermination Ribosomes for Reinitiation on GCN4 and Together with eIF3i Stimulates Linear Scanning. *Mol Cell Biol*, **30**, 4671-4686.
5. Chernoff, Y.O., Derkach, I.L. and Inge-Vechtomov, S.G. (1993) Multicopy SUP35 gene induces de-novo appearance of psi-like factors in the yeast Saccharomyces cerevisiae. *Curr Genet*, **24**, 268-270.
6. Bradley, M.E., Bagriantsev, S., Vishveshwara, N. and Liebman, S.W. (2003) Guanidine reduces stop codon read-through caused by missense mutations in SUP35 or SUP45. *Yeast*, **20**, 625-632.
7. Brachmann, C.B., Davies, A., Cost, G.J., Caputo, E., Li, J., Hieter, P. and Boeke, J.D. (1998) Designer deletion strains derived from Saccharomyces cerevisiae S288C: a useful set of strains and plasmids for PCR-mediated gene disruption and other applications. *Yeast*, **14**, 115-132.
8. Kurjan, J., Hall, B.D., Gillam, S. and Smith, M. (1980) Mutations at the yeast SUP4 tRNATyr locus: DNA sequence changes in mutants lacking suppressor activity. *Cell*, **20**, 701-709.
9. Beznosková, P., Cuchalová, L., Wagner, S., Shoemaker, C.J., Gunišová, S., Von der Haar, T. and Valášek, L.S. (2013) Translation initiation factors eIF3 and HCR1 control translation termination and stop codon read-through in yeast cells. *PLoS Genet*, **9**, e1003962.
10. Gietz, R.D. and Sugino, A. (1988) New yeast-Escherichia coli shuttle vectors constructed with in vitro mutagenized yeast genes lacking six-base pair restriction sites. *Gene*, **74**, 527-534.
11. Bidou, L., Stahl, G., Hatin, I., Namy, O., Rousset, J.P. and Farabaugh, P.J. (2000) Nonsense-mediated decay mutants do not affect programmed -1 frameshifting. *Rna*, **6**, 952-961.
12. Keeling, K.M., Lanier, J., Du, M., Salas-Marco, J., Gao, L., Kaenjak-Angeletti, A. and Bedwell, D.M. (2004) Leaky termination at premature stop codons antagonizes nonsense-mediated mRNA decay in S. cerevisiae. *RNA*, **10**, 691-703.
13. Asano, K., Phan, L., Anderson, J. and Hinnebusch, A.G. (1998) Complex formation by all five homologues of mammalian translation initiation factor 3 subunits from yeast *Saccharomyces cerevisiae*. *J Biol Chem*, **273**, 18573-18585.

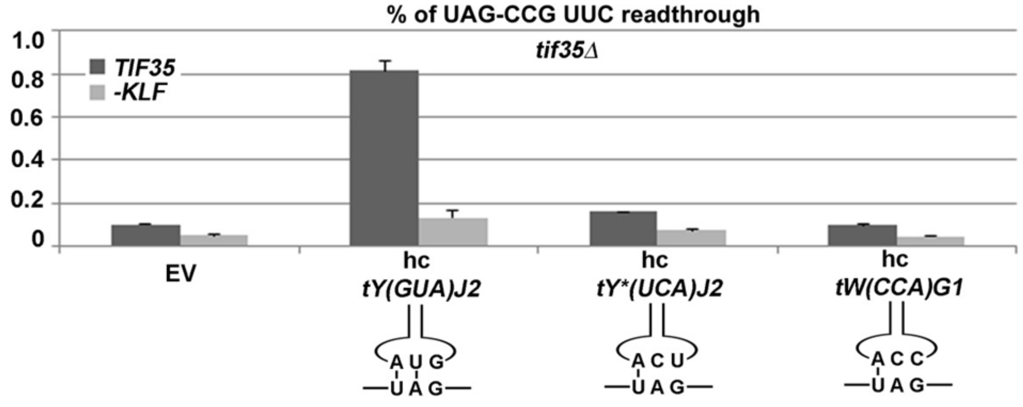

Supplementary Figure S1. eIF3 promotes incorporation of aminoacyl-tRNAs with a mismatch at the 3rd position only of all three stop codons. eIF3 enhances incorporation of the near-cognate tRNA also at the UAG stop codon with a mismatch at the 3rd position [tY(GUA)J2 – encoding tyrosine] but not with non-cognate tY*(UCA)J2 or tW(CCA)G1. The PBH140 derivatives bearing *TIF35* wt and *tif35-KLF* mutant alleles (generated as described in Figure 2) were transformed with empty vector (EV), hc tY(GUA)J2, hc tY*(UCA)J2 or hc tW(CCA)G1, and subsequently also with the readthrough construct YEp-R/T-UAGC-L, and the resulting transformants were grown and processed for stop codon readthrough measurements as described in Experimental Procedures.
